# Supplementary material for: The Role of Human Endogenous Retrovirus (HERV)-K119 env in THP-1 Monocytic Cell Differentiation
Source: Int J Mol Sci. 2023 Oct 25;24(21):15566. doi: 10.3390/ijms242115566 (PMC10648273; doi:10.3390/ijms242115566)
Supplement: Supplementary file 1 [file ijms-24-15566-s001.zip › ijms-2602387-supplementary.pdf]

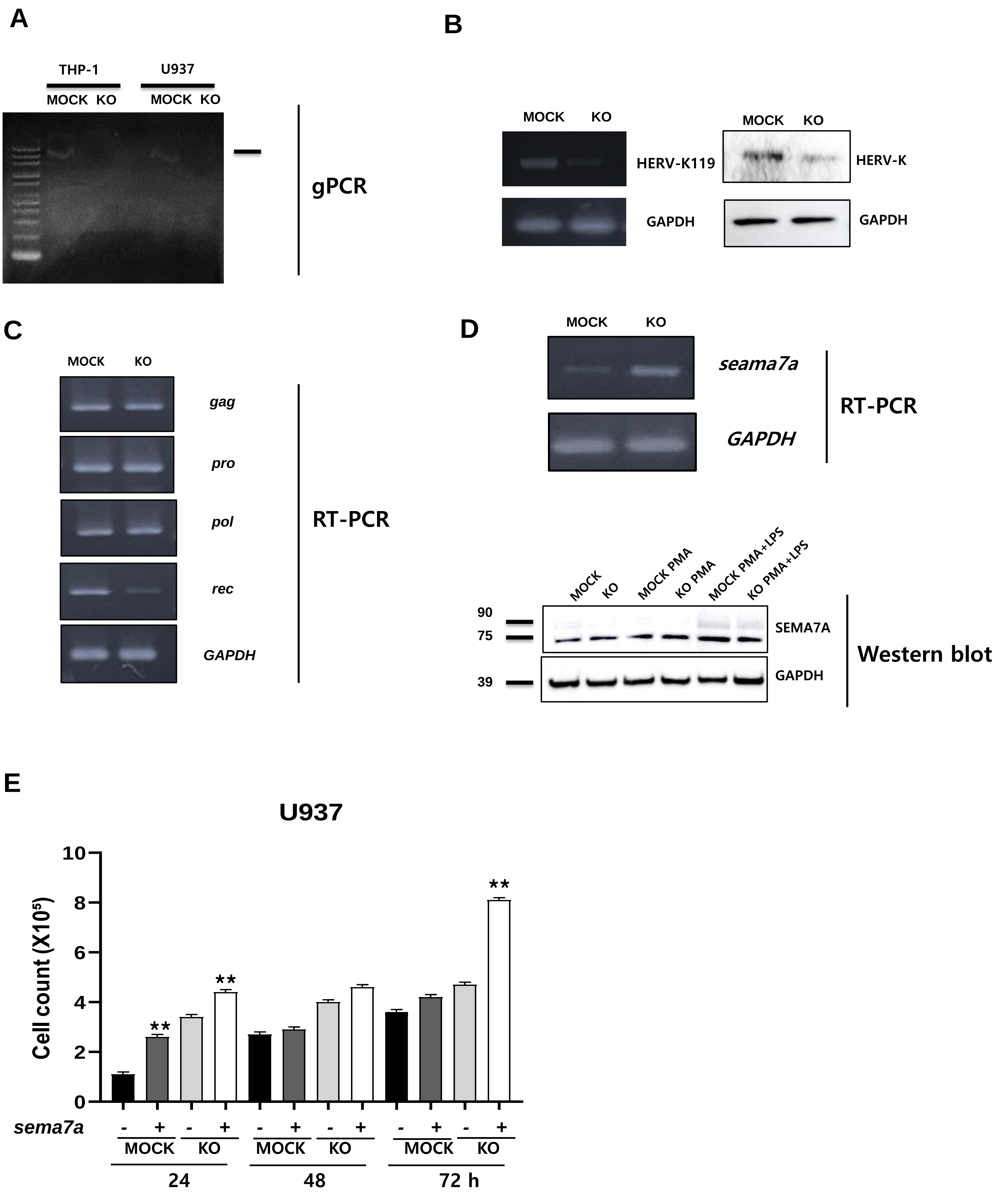

Supplementary Figure S1. HERV-K *env* KO in U937 monocytic cell.

(A) Knockout of HERV-K *env* gene in HERV-K119 region. Genomic polymerase chain reaction (gPCR) was performed for specific regions of HERV-K *env* gene to confirm its KO in U937 monocytic leukemia cells. (B) Expression of HERV-K *env* gene in HERV-K *env* KO U937 cells. The expression of HERV-K *env* at RNA and protein levels was analyzed using RT-PCR and Western blotting. (C) Expression of HERV-K119 *gag, pro, pol* and *rec* genes in U937 cells. (D) Expression of *sema7a* gene at RNA and protein levels. (E) Cell proliferation rate was significantly increased by over-expressing *sema7a* gene in U937 cells
